# Neighborhood disadvantage and firearm injury: does shooting location matter?

**DOI:** 10.1186/s40621-021-00304-2

**Published:** 2021-03-08

**Authors:** Kimberly Dalve, Emma Gause, Brianna Mills, Anthony S. Floyd, Frederick P. Rivara, Ali Rowhani-Rahbar

**Affiliations:** 1 Department of Epidemiology, School of Public Health, University of Washington, Hans Rosling Center for Population Health, 3980 15th Avenue NE, Box 351619, Seattle, WA 98195-7230 USA; 2 Firearm Injury & Policy Research Program, Harborview Injury Prevention & Research Center, University of Washington, 325 Ninth Avenue, Box 359960, Seattle, WA 98104 USA; 3 Alcohol and Drug Abuse Institute, University of Washington, 1107 NE 45th St., Suite 120, Box 354805, Seattle, WA 98105-4631 USA

**Keywords:** Firearm violence, Neighborhood disadvantage, Injury epidemiology

## Abstract

**Background:**

Firearm violence is a public health problem that disparately impacts areas of economic and social deprivation. Despite a growing literature on neighborhood characteristics and injury, few studies have examined the association between neighborhood disadvantage and fatal and nonfatal firearm assault using data on injury location. We conducted an ecological Bayesian spatial analysis examining neighborhood disadvantage as a social determinant of firearm injury in Seattle, Washington.

**Methods:**

Neighborhood disadvantage was measured using the National Neighborhood Data Archive disadvantage index. The index includes proportion of female-headed households with children, proportion of households with public assistance income, proportion of people with income below poverty in the past 12 months, and proportion of the civilian labor force aged 16 and older that are unemployed at the census tract level. Firearm injury counts included individuals with a documented assault-related gunshot wound identified from medical records and supplemented with the Gun Violence Archive between March 20, 2016 and December 31, 2018. Available addresses were geocoded to identify their point locations and then aggregated to the census tract level. Besag-York-Mollie (BYM2) Bayesian Poisson models were fit to the data to estimate the association between the index of neighborhood disadvantage and firearm injury count with a population offset within each census tract.

**Results:**

Neighborhood disadvantage was significantly associated with the count of firearm injury in both non-spatial and spatial models. For two census tracts that differed by 1 decile of neighborhood disadvantage, the number of firearm injuries was higher by 21.0% (95% credible interval: 10.5, 32.8%) in the group with higher neighborhood disadvantage. After accounting for spatial structure, there was still considerable residual spatial dependence with 53.3% (95% credible interval: 17.0, 87.3%) of the model variance being spatial. Additionally, we observed census tracts with higher disadvantage and lower count of firearm injury in communities with proximity to employment opportunities and targeted redevelopment, suggesting other contextual protective factors.

**Conclusions:**

Even after adjusting for socioeconomic factors, firearm injury research should investigate spatial clustering as independence cannot be able to be assumed. Future research should continue to examine potential contextual and environmental neighborhood determinants that could impact firearm injuries in urban communities.

## Background

Firearm violence displays a marked geographic distribution concentrated in certain areas of notable economic and social disparities. Researchers have been examining the connection between deprivation and violence for decades, using various measures of neighborhood disadvantage and violence such as violent crime (Baumer et al., 2003; Hsieh & Pugh, 1993; Lauritsen & White, 2001), and homicide (Jones-Webb & Wall, 2008). Considering firearm violence using a public health framework may identify structural risk factors to facilitate longer-term effects compared to interventions that solely focus on high-risk individuals (Branas et al., 2017).

Measures of poverty and disadvantage including income inequality (Rowhani-Rahbar et al., 2019), social capital, social mobility, and local welfare spending (Kim, 2019) have been found to be associated with firearm homicide across the United States. However, non-fatal firearm injuries make up the majority of firearm injuries and research has been limited by examining fatal firearm injury (Kalesan et al., 2017; Hipple et al., 2019). As fatality often depends on the anatomical area of injury (Beaman et al., 2000) and distance from trauma centers (Circo, 2019), relying on fatal firearm injury only may provide an incomplete understanding of the determinants of firearm injury.

In recent years, some investigators have been able to access fatal and non-fatal firearm assaults through hospital or law enforcement data. In Miami-Dade County, areas with a higher percentage of Black residents and higher percentage of single-parent households had significantly higher rates of firearm injury (Zebib et al., 2017). Firearm assaults were observed in areas with low household income in Philadelphia (Beard et al., 2017) and racial segregation in Massachusetts (Krieger et al., 2017). Nonfatal and fatal shootings in Indiana were concentrated in areas of disadvantage classified by percent unemployment, median household income, percent living in poverty, and percent female headed household (Magee, 2020). Poverty, segregation, and education were found to be predictors of firearm violence in urban California (Goin et al., 2018).

When non-fatal injury information is available, often residence is used as a proxy of location of injury. However, in King County, Washington where the city of Seattle is located, 75% of firearm assault injuries occurred in a census tract different from where the patient resided, and patients ages 18–34 had the greatest distances between injury location and residence (Mills et al., 2019). Understanding the association between neighborhood disadvantage of the injury location and risk of firearm injury can strengthen support for more structural prevention initiatives and is distinct from the risk associated with residence in an area of concentrated disadvantage.

We sought to expand the literature by examining location of both fatal and non-fatal shootings in relation to neighborhood disadvantage as a social determinant of health in the City of Seattle, Washington. We hypothesized that neighborhood disadvantage is associated with firearm assault injury, with neighborhoods at higher disadvantage experiencing greater risk of firearm injuries. This study adds to the growing literature examining neighborhood disadvantage and firearm violence in urban areas, specifically, and addresses potential limitations of prior research by employing methods that account for the non-independence of injuries across space and including both non-fatal and fatal firearm assault events at the location of injury.

## Methods

This was an ecological study using Bayesian spatial analysis conducted at the census tract level in Seattle, Washington. A census tract is a subdivision of a county or similar entity and contains between 1200 and 8000 people. The city of Seattle consists of 142 census tracts including partial tracts.

### Data sources

Two main data sources were used to identify firearm assault injuries within Seattle between March 20, 2016 and December 31, 2018. First, patients with a documented assault-related gunshot wound were identified from medical records at Harborview Medical Center (the regional Level I trauma center). For these analyses, we used data collected in another research project that began March 20, 2016 and ended enrollment on December 31, 2018*.* Since individuals who did not survive long enough to be transported to the hospital were missing from medical records, the Gun Violence Archive (GVA) was used to supplement additional cases of firearm assault injury in Seattle. GVA is an open-source dataset that uses over 7500 sources to document incidents of gun violence in the United States (Gun Violence Archive, 2020). GVA includes both fatal and non-fatal incidents and includes a variety of coded variables and links to additional source information, typically from news sites, providing many demographic and circumstance details. When someone appeared in both datasets (i.e. someone sustained an injury and was treated in the hospital but did not survive), information from the hospital data was used. Firearm assault injury was defined as an intentional interpersonal injury involving firearms. This included assaults and homicides. Unintentional, undetermined intent, and self-inflicted firearm injuries were excluded from the analysis.

Among hospitalized patients, injury locations were identified based on hospital records. For hospitalized patients missing complete addresses, internet news archives were used to determine injury location. Injury locations are provided within GVA as addresses. All available addresses were geocoded to identify their point locations and then aggregated to the census tract level.

Neighborhood disadvantage from the National Neighborhood Data Archive (NaNDA) at the census tract level was then merged to the firearm injury file. The NaNDA socioeconomic status and demographic characteristics of census tracts uses the American Community Survey 5-year estimates from 2013 to 2017 to provide information by census tracts for the United States and Puerto Rico (Melendez et al., 2020). The index was developed using principal factor analysis (Clarke et al., 2014). Among the census measures included in the analysis, there were three factor loadings interpreted as neighborhood disadvantage, neighborhood affluence, and ethnic and immigrant concentration. The current study used the four-indicator neighborhood disadvantage index which includes proportion of female-headed families with children, proportion of households with public assistance income, proportion of people with income below poverty in the past 12 months, and proportion of the civilian labor force aged 16 and older that were unemployed. These proportions were averaged for each census tract.

For the current study, these averaged proportions were then sorted at the state level (Washington) and ranked in deciles to create a scale 1–10, with 1 being the least disadvantaged and 10 being the most disadvantaged. This creation of a rank-type format allows for easier interpretation and comparison between neighborhoods. Annual population estimates for Seattle census tracts for 2016–2018 were obtained from the Washington State Office of Financial Management (Washington State Office of Financial Management, 2019).

### Statistical analysis

Bayesian Poisson models were fit to the data to estimate the association between the index of neighborhood disadvantage and firearm injury count with a population offset within a census tract. The first model was a non-spatial model with independent and identically distributed (IID) random effects. The IID model did not account for the neighboring structure but smooths outliers towards the center of the data. The second model was an Intrinsic Conditional Auto-Regressive (ICAR) Besag-York-Mollie (BYM2) model which incorporated the spatial structure of neighboring data by including both IID and spatial random effects (Riebler et al., 2016). Thereby, the model included both normally distributed error terms as well as a spatial component estimated as the weighted mean of the firearm injuries in each area’s neighboring census tracts. In the spatial model, neighbors were defined as any contiguous census tracts that shared a common border. Annual population estimates of the census tract for the same time period of the study (2016–2018) were log transformed and included in the models as an offset. Both models used integrated nested Laplace approximations to estimate posterior distributions (Wakefield, 2007). Additionally, as a sensitivity analysis, a final BYM2 model was fit without including the neighborhood disadvantage index to assess the proportion of the variance that was spatial when no explanatory variables were included. All analyses were conducted in the statistical programming language R (www.r-project.org) using the INLA package (www.r-inla.org).

### Ethical review

This study was approved by the University of Washington Human Subjects Division (Institutional Review Board; STUDY00000852).

## Results

From March 20, 2016 through December 31, 2018, there were 219 firearm assault injuries identified in Seattle of which 191 were able to be geocoded by place of occurrence (87.2%). Of the persons experiencing these injuries, the average age was 30.8 years (SD: 11.9) and 82.7% were male. 25.1% of injuries were determined to be fatal by hospital records or according to GVA sources.

**Fig. 1:**
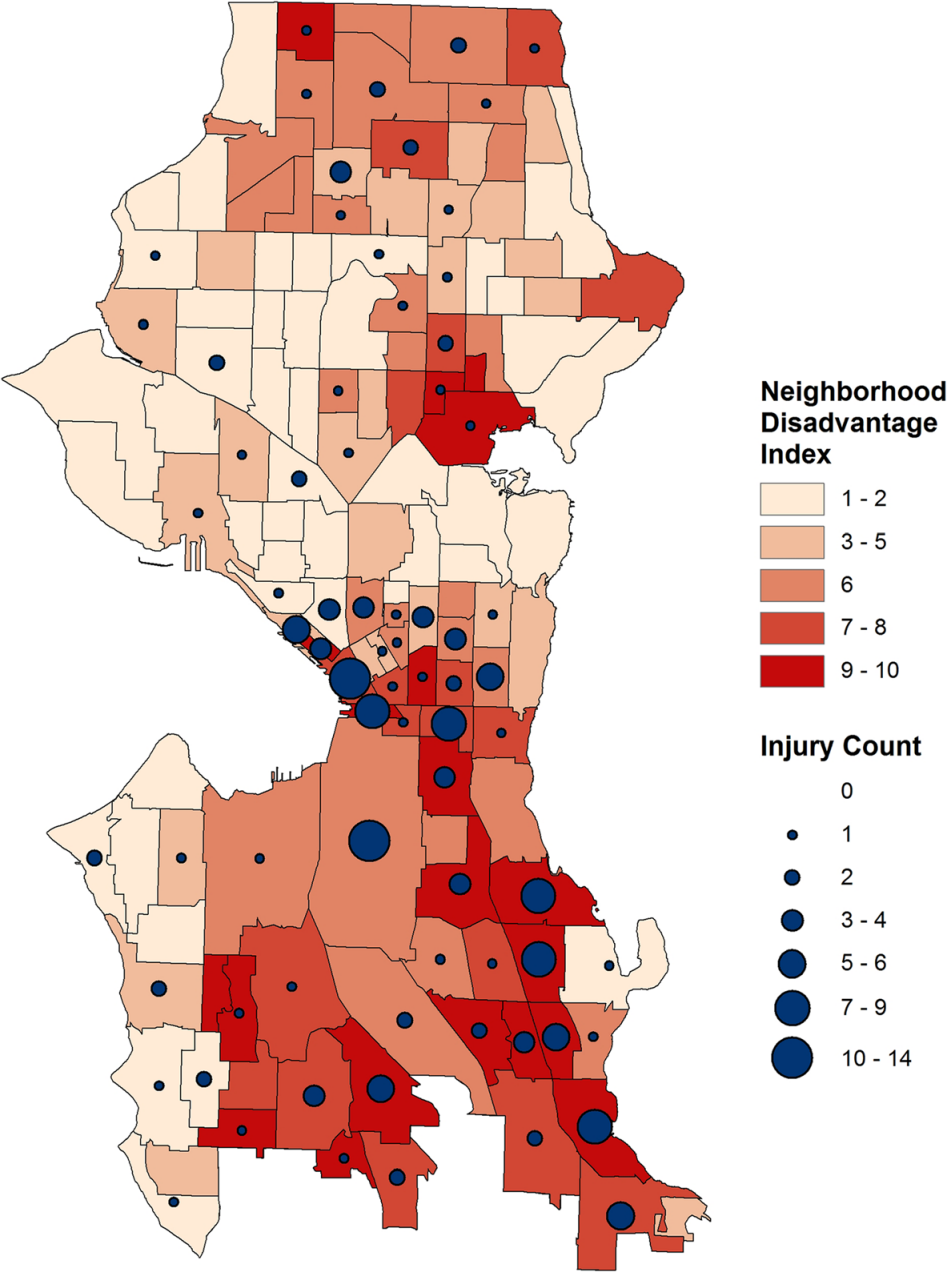
Map of Seattle Census Tracts. Color scale by disadvantage index with circles sized by number of firearm assault injury events and centered in the census tract

Among Seattle’s 142 census tracts, the average disadvantage index was 4.76 (SD: 2.97) (Fig. 1). Over the study period, the number of injuries within a census tract ranged from 0 to 14 per census tract, with an average of 1.4 injuries. 63 tracts experienced no firearm injuries; all firearm assault injuries (*n* = 191) occurred in 55.6% of Seattle’s census tracts (*n* = 79). Half (*n* = 87) of the firearm assault injuries occurred in 14 (9.9%) census tracts.

In the IID model, for two census tracts that differ in neighborhood disadvantage by 1 decile, the number of firearm injuries was expected to be higher by 27.6% (95% credible interval: 18.1, 39.0%) in the tract with the higher neighborhood disadvantage.

In the BYM2 spatial model, higher neighborhood disadvantage was associated with more firearm injuries, but the results were somewhat attenuated. For two census tracts that differed by 1 decile of neighborhood disadvantage, the number of firearm injuries was expected to be higher by 21.0% (95% credible interval: 10.5, 32.8%) in the group with higher neighborhood disadvantage. However, even after accounting for spatial structure by contiguous neighbors, there was still considerable residual spatial dependence with 53.3% (95% credible interval: 17.0, 87.3%) of the model variance being spatial. This suggests the presence of residual confounding by location. As a sensitivity analysis, another BYM2 model was fit without including the neighborhood disadvantage index to compare the proportion of the variance that was explained by including a measure of disadvantage in the model. When examining the spatial dependence of firearm assault injuries without the disadvantage index, 75.2% (95% credible interval: 37.1, 95.2%) of the model variance was spatial.

**Fig. 2:**
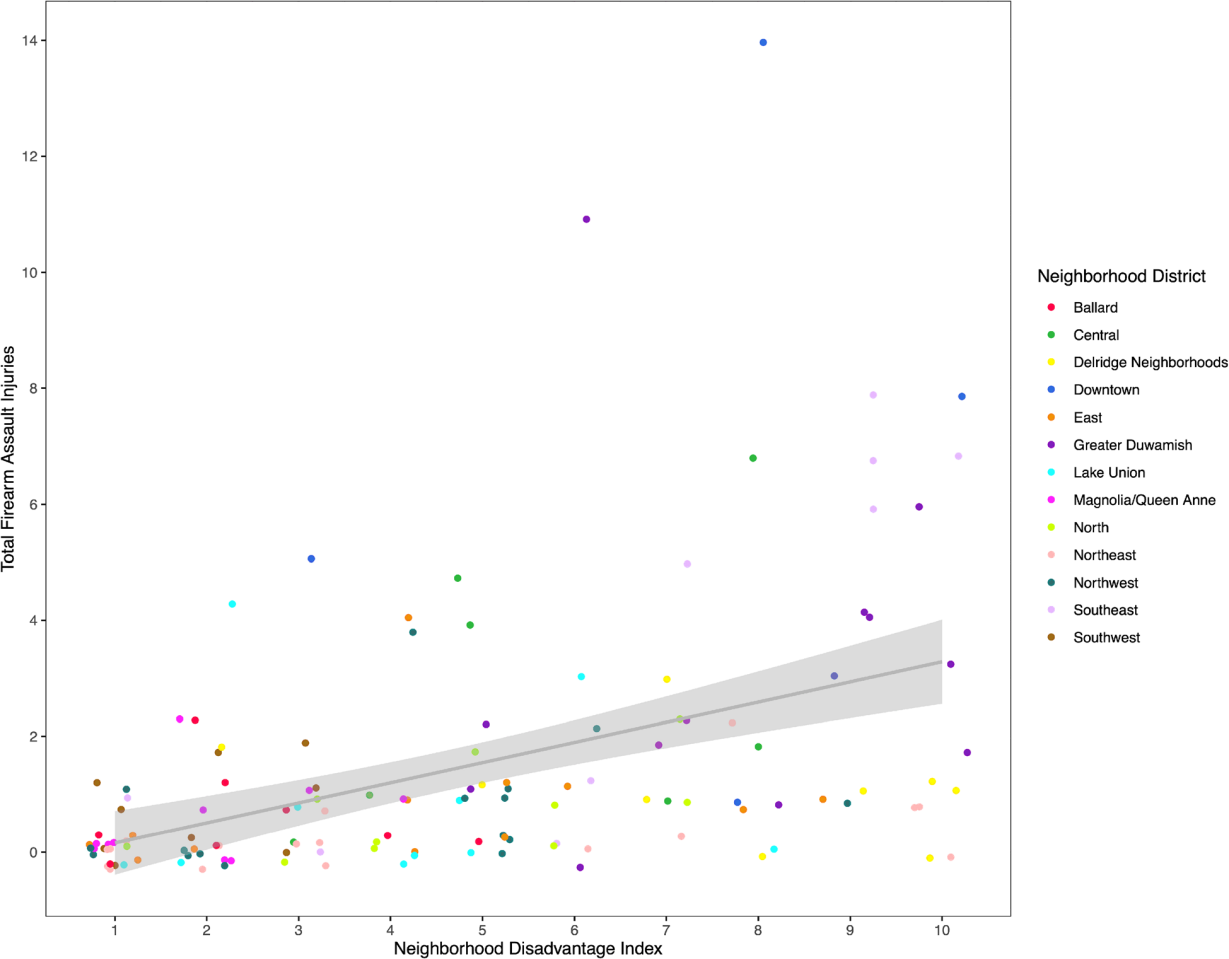
Scatterplot of neighborhood disadvantage index and firearm assault injuries of the census tract. Linear fit line for exploratory characterization, color denotes Neighborhood District name

**Fig. 3:**
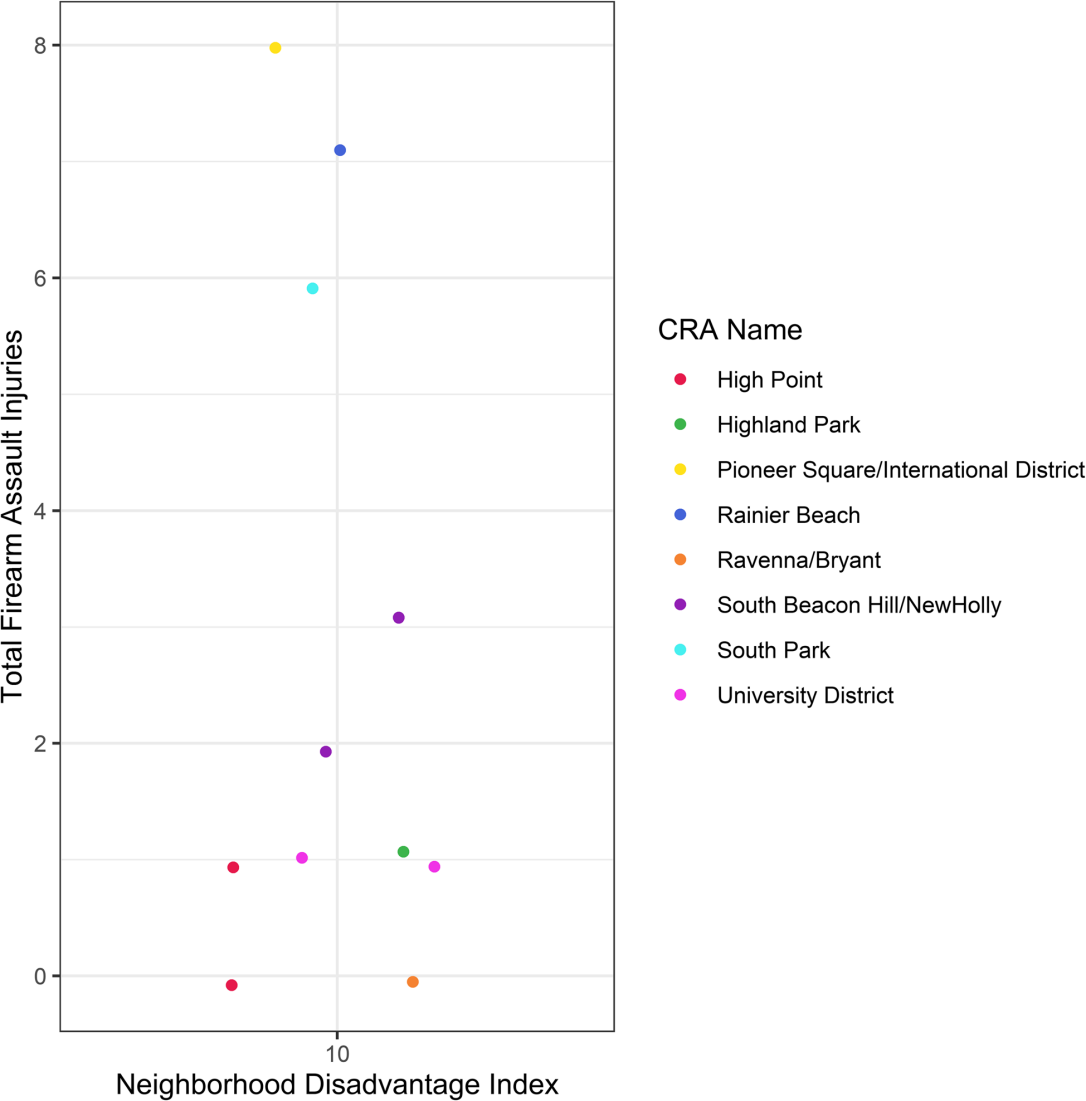
Firearm assault injury count for census tracts with a neighborhood disadvantage index of 10. Color denotes the Community Reporting Area (CRA) name

In visually inspecting the association between neighborhood disadvantage index and firearm injury (Fig. 2), we observed areas with high neighborhood disadvantage and low firearm injury count. Several of these data points were from the Delridge neighborhood district, specifically in the community reporting areas of High Point and Highland Park (Fig. 3).

## Discussion

This study demonstrates the importance of considering the geographic context when studying social determinants of firearm violence. Geographic analysis of crime has been prominent in Seattle with micro-place analysis of crime examining street segments (Weisburd et al., 2004). Even when considering larger areas such as census tracts and examining firearm violence specifically, we found a similar clustered pattern where a few areas contributed to the majority of events. When examining the association of neighborhood deprivation with firearm injuries, neighborhood disadvantage had a significant association in both the non-spatial and spatial models, though the results were attenuated when space was included. Firearm injury research should investigate spatial clustering when performing analyses as independence may not be able to be assumed even after adjusting for socioeconomic factors. This is particularly important for studying interpersonal outcomes, such as assault, where social proximity may be as important as geographic distance (Papachristos et al., 2015).

In using firearm injury location data as opposed to residence location, we were able to determine that where an injury occurs is related to the neighborhood’s deprivation; however, even after accounting for the influence of neighboring tracts, a large proportion of the variance was spatial. Together these results suggest that areas with high firearm injury rates tend to cluster together and the influence of injuries in neighboring tracts has an effect on the outcome of firearm injuries in a location above and beyond the census tract’s level of deprivation. Future investigations should explore other potential explanatory variables that might be able to explain some of this residual variability and explore why proximate tracts have similar firearm injury rates even after accounting for the effect of neighborhood deprivation.

There may be other contextual factors that affect firearm injuries in these locations including environmental factors such as greenspace and vacant lots (Bogar & Beyer, 2016, Branas et al., 2016, Moyer et al., 2018). Neighboring census-tracts may also be similar in other characteristics associated with firearm violence such as education (Goin et al., 2018), gun ownership (Monuteaux et al., 2015), and alcohol outlet density (Furr-Holden et al. 2019; Mair et al. 2013). These factors may be potential confounders in the relationship between neighborhood disadvantage and firearm injury or could be part of the pathway from neighborhood disadvantage to firearm violence, perpetuating a cycle of disadvantage and firearm violence. These potential feedback effects could be explored in future work.

We observed areas with high disadvantage and low firearm injury suggesting future research should examine potential protective factors or resiliency at a neighborhood level such as social cohesion and capital (Lochner et al., 2003; Sampson et al., 1997). These were noted in communities of working-class families with historical ties to employment opportunities, Highland Park which is located near Boeing Field and the Industrial District, and redevelopment. High Point was originally developed during World War II as government housing. From 2003 to 2010, Seattle Housing Authority redeveloped the area into a mixed-income neighborhood with attention to environmental sustainability and community engagement. In Duval County, Florida, areas with high neighborhood deprivation but low firearm injury were also noted to have been targeted by community revitalization efforts (Abaza et al., 2020). Further work examining neighborhoods with high deprivation and low firearm injury may provide additional information on protective factors that could be strengthened by community organizations. Community revitalization efforts that impact neighborhood disadvantage may also reduce firearm injuries. Community revitalization may be impacting the built-environment and increasing social capital, strengthening informal social controls (Sampson et al., 1997).

The findings from the current study can be used to assist in strategic planning of firearm prevention efforts. In 2009, the Seattle Youth Violence Prevention Initiative was initiated to reduce youth violence and included various programming for at-risk youth ages 12–17 as well as street outreach services (City of Seattle Office of City Auditor, 2015). This initiative was unable to be evaluated due to lack of defined goals and data management systems (City of Seattle Office of City Auditor, 2014). Firearm prevention programs may consider integrating neighborhood disadvantage reduction as a part of primary prevention.

### Limitations

The NaNDA measure of neighborhood disadvantage features items commonly seen in the violence and health literature (Clarke et al., 2014). However, this index may not fully capture the structural context of deprivation associated with firearm violence. This study used residential population of each census tract as the population offset. However, notably in urban areas, the population at risk may greatly fluctuate in different timings throughout the day based on routine activities such as school, work, transportation hubs, and nightlife (Walker et al., 2014). An area may have a large number of non-residents that may differ in sociodemographic characteristics who spend time in the census tract. The current analysis includes all assault injuries; however, there may be important distinctions depending on type of assault such as domestic violence and gang violence. Future research should examine if the association of neighborhood disadvantage and firearm injury differs by these intents.

Additionally, the reliance on administrative boundaries such as census tract for which these data are available, may not capture the identity of a neighborhood. Geographic level is an important consideration (Hipp, 2007; Schnell et al., 2017; Mair et al., 2020) and other units of aggregation were considered such as census block group. However, with the number of firearm assault injury events, we believed aggregating smaller than the census tract may produce spurious findings due to the high proportion of zero incidence tracts at smaller geographic levels (Wakefield, 2004). Though we believe census tract was the relevant scale to represent the neighborhood context, more research understanding how certain theories of violence operate at different spatial scales is needed (Boessen & Hipp, 2015). In addition to the implications of these findings on community prevention efforts, the study highlights the need for available measurements at the neighborhood level of such protective factors, potential confounders, and modifiers. Measurements of income inequality, racial segregation and discrimination, gun ownership, and social capital and cohesion at smaller-scale geographies may further elucidate the relationship between neighborhood disadvantage and firearm injury.

The current study was an ecological analysis with exposure and outcome measured close together or concurrently in time, neighborhood deprivation measures from 2013 to 2017 and firearm injury from 2016 to 2018. Future research should consider additional time periods of neighborhood deprivation that could have differing effects on firearm injury or demonstrate different pathways such as the effect of lifetime of experience, historical deprivation (Benns et al., 2020; Jacoby et al., 2018), and neighborhood changes, including gentrification (Schnake-Mahl et al., 2020). In a prior study examining the effects of gentrification on crime in Seattle from 1982 to 2000, it was not found to be statistically significantly associated with violent crime (Kreager, Lyons, & Hays, 2011). However, as gentrification was observed to change from the small-scale investments in the 1980s to larger corporate investors and urban renewal programs in the 1990s, neighborhood changes in the recent decades may by qualitatively different and could influence firearm violence.

Lastly, this study features findings from one city. The firearm homicide rate in Seattle and King County decreased from 2007 to 2010, and by 2016 increased to the rate observed in 2000 (Public Health — Seattle & King County, 2019). According to data from the King County Prosecuting Attorney’s Office, in 2017, there was an increase in firearm homicides and non-fatal injuries across the county while 2018 showed decreases that more closely resembled 2016 (King County Prosecuting Attorney’s Office, 2019). The results may not be generalizable to other cities but adds to the previous literature of neighborhood disadvantage and firearm violence in urban areas.

## Conclusion

Firearm injuries depict geographic patterns that are associated with neighborhood disadvantage in Seattle, WA. Despite prior research on neighborhood disadvantage and violence, fewer studies have examined the association between neighborhood disadvantage and fatal and non-fatal firearm assault violence using data on injury location. As spatial methods techniques advance and become more accessible, these methodological considerations could have implications for findings and therefore, prevention recommendations.

In addition to using spatial analysis to identify areas with high burden of violence, the association of neighborhood disadvantage with firearm assault injury should consider addressing and evaluating programs and practices that remedy concentrated disadvantage as reducing neighborhood deprivation could reduce firearm injuries as well. Community-level programs and practices can consider unique opportunities for place-based firearm injury and violence prevention that would otherwise be lost or misallocated. However, as neighborhood disadvantage is not the only spatial measure affecting firearm violence, future research should consider other neighborhood contextual factors that could serve as risk or protective factors.

## Abbreviations

GVA: Gun Violence Archive
NaNDA: National Neighborhood Data Archive
IID: independent and identically distributed
ICAR: Intrinsic Conditional Auto-Regressive
BYM2: Re-parameterized Besag-York-Mollie
